# Dental Morphology in Restorative Dentistry: A Pilot Study on Morphological Consistency and Variability in Human Upper First Molars

**DOI:** 10.3390/dj13030122

**Published:** 2025-03-11

**Authors:** Gregorio Oxilia, Mauro Tomasella, Alberto Cecere

**Affiliations:** 1 Department of Medicine and Surgery, “LUM” Giuseppe Degennaro Casamassima, Casamassima, 70010 Bari, Italy; 2 Dental Laboratory (Dentalprotesi Srl), Via S. Caterina da Siena 9, Conegliano, 31015 Treviso, Italy; mtomasella67@gmail.com; 3 Dental Laboratory, Via G. Pascoli 2/c, Tivoli Terme, 00011 Rome, Italy; cecerlab@gmail.com

**Keywords:** contralateral teeth, dental variation, digital technologies, crown morphology, upper first molars (M1), dental morphology

## Abstract

**Background:** Geometric morphometric analysis, a methodology traditionally used in evolutionary studies, offers unprecedented precision in quantifying the morphological traits of human organs and tissues by identifying specific anatomical landmarks. Despite its potential, this approach has not yet been applied in medical or dental research. **Materials and Methods:** This pilot study analyzed 76 permanent upper first molars (M1) from Italian individuals (38 males and 38 females), selected for their completeness, minimal wear (stage 1), and absence of pathological conditions or non-metric traits. Each individual contributed two contralateral molars: the left molar (Group A) and the right molar (Group B). The molars were scanned using a Sinergia Scan Advanced Plus optical scanner with 5 μm accuracy. The scans were processed with the Dental Scan 7.0 software to generate high-resolution STL files, followed by refinement in Meshlab to preserve the morphological integrity of the 3D models. The geometric morphometric analysis was performed using the Viewbox software, thereby enabling the placement of anatomical landmarks and semilandmarks to quantify and compare molar morphology with exceptional precision. **Results:** The analysis confirmed that contralateral molars exhibit high morphological similarity, with significantly smaller variability compared to molars from different individuals. Among the specific traits analyzed, the distolingual cusp (hypocone) showed the greatest variation, followed by the mesiolingual cusp (protocone). No significant differences were observed between males and females in the degree of similarity between contralateral molars. **Conclusions:** This pilot study highlights the potential of geometric morphometric approaches to enhance our understanding of the dental variation between sexes and across human populations, thereby improving clinical applications and advancing toward personalized medicine.

## 1. Introduction

The study of contralateral teeth has become an increasingly important area of research, as analyzing the similarities and differences between teeth from the same individual provides valuable insights for both clinical applications and dental research. Numerous studies have confirmed a high degree of similarity (in length, width, dentinal thickness, and root canal configurations), which is likely due to shared developmental pathways and genetic factors (for example, [1,2,3,4,5,6]). some scholars [7] used micro-computed tomography (micro-CT) to analyze contralateral maxillary second molars, reporting a significant similarity in linear measurements and root canal configurations, with symmetry rates reaching 94.4% for palatal canals. Similarly, others [8] demonstrated substantial symmetry in contralateral premolars, while noting some variability in the apical portion, such as the distribution of accessory canals. Olthers [6] confirmed a high degree of symmetry in mandibular incisors, although greater variability was observed in the apical third and differences in root length between central and lateral incisors.

Despite these findings, significant gaps remain in the literature, particularly regarding the methods used to analyze in detail the morphological variations of dental crowns. Specifically, in the medical field, and more precisely in dentistry, innovative methodologies such as geometric morphometrics (GMMs) have been underutilized. These digital methods, however, have been successfully applied in evolutionary anthropology [9,10,11,12]. This approach allows for the precise quantification of morphological differences, offering new insights into the genetic differences between populations or races. Tooth morphology may serve as an indicator of genetic disturbances or evolutionary adaptations, and occlusal features such as cusps and grooves play a key role in identifying these patterns. Additionally, the morphological variability (non-metric traits; [13]) and abnormalities in teeth (such as increased wear patterns [14]) have significant clinical implications, including challenges in band placement, increased plaque accumulation due to abnormal fissure morphology, a higher risk of caries in deep pits, occlusal disturbances caused by an abnormal cusp location, and difficulties in restorative, surgical, and endodontic procedures. The maxillary first molar, the largest of all maxillary molars, is particularly important in mastication and plays a critical role in dental morphology studies. It is also a key tooth for gender differentiation due to its morphometric characteristics. Its high anchorage value makes it essential in orthodontic treatments, further emphasizing the need to understand its variations in cusp morphology and overall form. Moreover, the human maxillary first molar provides important clues about evolution and is functionally significant in both clinical and anthropological contexts. In this study, upper first molars from 76 adult (38 female and 38 male) Italian dental casts were analyzed using GMM. This research, the first in a series, aims to quantify the morphological differences between contralateral molars from the same individual and molars from different individuals. It focuses on analyzing the occlusal morphological differences of permanent teeth, starting with the maxillary first molar—a cornerstone of the dentition and a complex tooth that plays a crucial role in mastication. The study explores the potential of geometric morphometric analysis, a methodology traditionally used in evolutionary studies, to quantify the morphological traits of human teeth with exceptional precision. By applying this innovative approach to contralateral molars, the research seeks to identify and quantify the variability in specific anatomical features, such as cusps, ridges, and grooves, providing detailed insights into dental morphology. The ultimate goal is to establish a methodological foundation for improving the accuracy of dental prosthetic design and advancing personalized medicine by leveraging precise, individualized morphological data.

## 2. Materials and Methods

The sample consists of permanent upper first molars (M1) with similar wear stage (equal to 1 based on Molnar [1]) of 76 Italian individuals comprising both males (38 individuals) and females (38 individuals). The individuals analyzed in this study are organized into two groups, with each individual contributing two contralateral upper first molars, labeled as Group A and Group B. Group A represents the left molar from the individual, while Group B represents the contralateral (right molar) from the same individual. The individuals in this study were selected based on the availability of complete and well-preserved first upper molars, with no signs of major pathological conditions, extensive wear, or non-metric traits that could affect the analysis. None of the individuals presented general diseases, and the antagonist teeth showed no alterations, ensuring that the morphology of the analyzed upper molars was not affected. Additionally, all individuals exhibited a Class I occlusion, further confirming the absence of occlusal or morphological alterations in the first upper molars. The sample includes individuals with known sex, but no specific information on age or provenance was included in the dataset provided. The dataset ensures a balanced representation of males and females, with each individual contributing a pair of first upper molars for the comparison.

### 2.1. Data Acquisition

A total of 76 upper first molars, acquired from dental casts of recent individuals (19 females and 19 males and their mirrored contralateral tooth), were scanned at the Dentalprotesi S.r.l. laboratory using a Sinergia Scan Advanced Plus scanner (Sinergia S.r.l., Milan, Italy). This optical scanner operates with a 5-axis system and a tilting plate (−15° to +25°) for enhanced imaging acquisition. Equipped with two cameras, it enabled the capture of surface texture details. The scanning process for an entire arch takes 12–15 s, utilizing the reference rim technique (photogrammetric scanning). The accuracy of the scanner is 5 μm, with repeatability at 2 μm and a resolution of 5 μm, adhering to ISO 12836 standards [15].

The digital reconstruction of the molars was performed using the Dental Scan 7.0 software (Open Technologies S.r.l., Brescia, Italy) to facilitate the acquisition and alignment of point clouds, as well as the generation of polygonal surfaces to obtain digital reconstructions of highly detailed STL files. Afterwards, the Meshlab software version 2023.12 (Visual Computing Lab-ISTI-CNR, Pisa, Italy) was employed for post-processing, which involved refining the 3D models, optimizing the triangle mesh, and removing any artifacts or imperfections, while preserving the morphological integrity of the original dental structures. Finally, a geometric morphometric analysis was carried out using the Viewbox software version 4.1.4.0 (trial version, dHAL Software, Kifissia, Greece), which enabled the placement of landmarks and semilandmarks that are necessary for the detailed morphometric comparison.

### 2.2. Geometric Morphometrics

**Figure 1:**
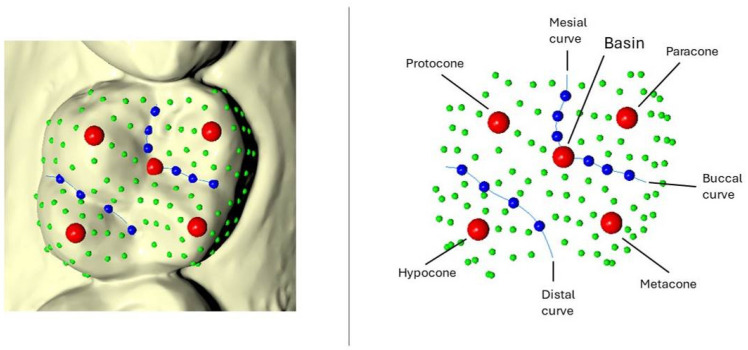
A template with landmarks (red), curve and surface semilandmarks (blue and green, respectively) digitized on a dental crown.

The upper first molars were analyzed using 3D landmark-based geometric morphometric (GM) methods [16,17], which allow for the identification of morphological traits rather than focusing solely on geometric patterns and volumes. A 3D template consisting of 115 semilandmarks—5 landmarks, 10 curve semilandmarks, and 100 surface semilandmarks—was created for the upper first molars (Figure 1). The landmarks were placed at key anatomical points on the molar to capture its overall shape. Curve semilandmarks were positioned along the cervical margin of the molar on both the buccal and palatal sides. Surface semilandmarks were distributed evenly across the occlusal surface to capture the detailed morphology of the crown. The landmarks were manually digitized using specialized 3D software, and the curve semilandmarks were spaced at 20% intervals along the curves, ensuring the accurate representation of the molar’s shape. The surface semilandmarks were placed to provide a comprehensive representation of the tooth’s occlusal topography, including the cusps and grooves, by following the natural curvature of the molar’s surface.

The semilandmarks were allowed to slide along their respective curves and surfaces using a thin-plate spline (TPS) algorithm, minimizing the bending energy between the template and the target configurations [18]. This sliding process ensures that semilandmarks are geometrically homologous across all individuals, in alignment with standard GM practices [19].

**Figure 2:**
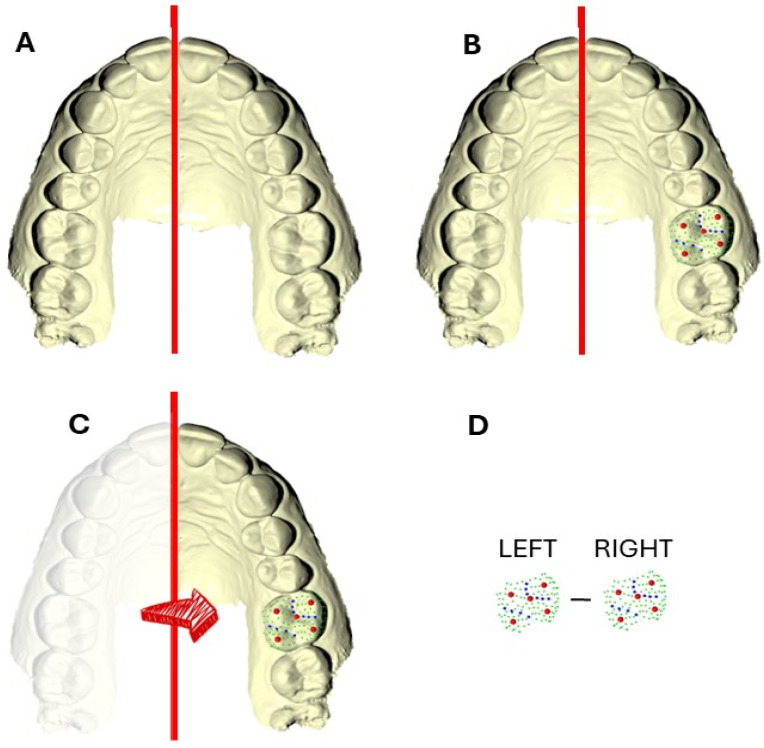
(**A**) Midsagittal plane (red line) used as a reference for the alignment of dental structures. (**B**) Placement of the geometric template on the left upper first molar. (**C**) Mirroring of the right hemimaxilla to align with the midsagittal plane for comparison. (**D**) Contralateral comparison of left and right first upper molars using semilandmarks, showing the potential morphological differences in their occlusal surfaces. Landmarks (red circles), line curves and curve semilandmarks (blue lines and circles, respectively), and surface semilandmarks (green circles) digitized on a dental crown.

In order to obtain geometrically homologous surface semilandmarks in the contralateral tooth, the complete digital dental cast was first considered (Figure 2A). The template was initially applied to the left upper first molar (Figure 2B). To accurately compare the morphology of the contralateral tooth, the right hemimaxilla was mirrored (Figure 2C) using the midsagittal plane, which passes through the incisal midline and the junction point of the transverse palatal suture. This plane was established as perpendicular to the occlusal reference plane, following the methodology outlined by Oxilia et al. [9]

The mirroring process allowed for the correct application of the template to the right molar, enabling the semilandmarks to slide along the curves (curve semilandmarks) and across the surface (surface semilandmarks). This process minimized the thin-plate spline (TPS) bending energy between the template and the mirrored copy [18], ensuring that the semilandmarks could be considered geometrically homologous [19]. It also facilitated the comparison of points on the occlusal surface, allowing for the identification of potential morphological differences (Figure 2D).

### 2.3. Statistical Analysis

After obtaining the post-GPA (Generalized Procrustes Analysis) coordinates for each upper first molar, the dataset was prepared for statistical analysis. The normality of the 3D coordinate data (X, Y, Z) was first evaluated using the Shapiro-Wilk test, followed by an assessment of variance with Levene’s test. These analyses helped determine whether parametric or non-parametric methods would be appropriate for the analysis. In addition, the variance along each axis was calculated to examine the spread of values across the different dimensions. Since the Shapiro-Wilk and Levene’s tests indicated that the data did not meet the assumptions of normality for certain dimensions, non-parametric methods were selected for all subsequent analyses to ensure robustness.

First, we investigated whether there was a significant difference between male and female molars, allowing us to pool the data into a single group for subsequent analyses, thus eliminating the need to account for gender-based distinctions when examining shape variability. A Procrustes alignment was applied to standardize the morphological configurations of the molar shapes, enabling direct comparisons between contralateral molars (left and right, labeled A and B) from the same individual, as well as comparisons between molars from different individuals. The Procrustes distance was used as a quantitative measure of morphological disparity between pairs of molars, providing a consistent metric for evaluating shape differences.

To assess intra-individual morphological similarity, the Euclidean distances were calculated between the contralateral molars (A vs. B) of each individual. These intra-individual distances were then compared with the Euclidean distances between molars from different individuals, measuring inter-individual variability. By comparing these two sets of distances, we aimed to determine whether molars from the same individual exhibited greater morphological similarity than molars from different individuals. For the statistical comparison of intra- and inter-individual distances, the normality and variance of the distance distributions were once again assessed using the Shapiro-Wilk and Levene’s tests. Given that the distributions did not meet the assumption of normality and variance, the non-parametric Wilcoxon signed-rank test was employed. This test allowed us to statistically evaluate whether the distances between contralateral molars of the same individual were significantly smaller than the distances between molars of different individuals.

To further explore the degree of morphological differences between contralateral molars and how the occlusal surface varies within individuals, we analyzed the variation in the coordinates of 115 landmarks positioned on the occlusal surface. These landmarks were obtained from the left and right first upper molars of 38 individuals. The Euclidean distances between corresponding landmarks on the left and right molars were calculated for each individual, and the mean variation was computed for each landmark across the sample.

Finally, to investigate the potential differences in morphological similarity between the two groups (females and males) considered separately, we calculated the Euclidean distances between contralateral molars for each individual, analyzing males and females separately. These intra-individual distances were compared between the groups to determine if one group exhibited a greater degree of similarity between its contralateral molars. A two-sample *t*-test was performed, as both the assumptions of normality (Shapiro-Wilk test) and equality of variances (Levene’s test) for the Euclidean distances were satisfied.

## 3. Results

**Figure 3:**
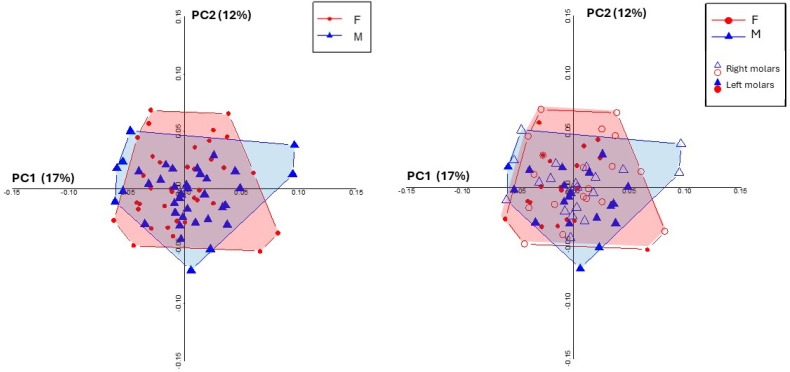
Principal component analysis (PCA) of the left and right first upper molars. The graph shows PC1 and PC2 axes for males (M) and females (F), with blue triangles representing male individuals and red circles representing female individuals. Left molars are depicted by filled symbols, while right molars are indicated by hollow symbols. The polygons represent the 95% confidence intervals for males (blue) and females (red), showing an overlap in the shape variation between sexes and sides.

The results of the Wilcoxon rank-sum test (PC1: W = 0.675, *p*-value = 0.500; PC2: W = 0.977, *p*-value = 0.329) indicated no significant difference between male and female molars (Figure 3). This allowed for the pooling of data into a single group for subsequent analyses, removing the need to account for gender-based distinctions when examining shape variability.

The primary objective of the study was to assess whether the molars from the same individual were more morphologically similar than those from different individuals. To achieve this, we calculated the Euclidean distances between the contralateral molars (left molar vs. right molar) of each individual, representing intra-individual variability. In parallel, the Euclidean distances between the molars of different individuals were computed to capture inter-individual variability.

The mean distance between contralateral molars from the same individual was 0.035, while the mean distance between molars from different individuals was 0.052. These initial findings suggested that the molars of the same individual were more morphologically similar than those from different individuals. To statistically validate this observation, a Wilcoxon signed-rank test was applied to compare the intra-individual and inter-individual distances. The analysis yielded a significant result (W = 121.0, *p* = 0.00015), confirming that the distances between contralateral molars of the same individual were significantly smaller than the distances between molars from different individuals.

Despite the overall similarity, the analysis of differences between contralateral molars revealed an average variation across the coordinates of the landmarks on the occlusal surface. A total of 115 landmarks on the left and right molars were compared for each of the 38 individuals. Among these landmarks, the palatal surface of the distolingual cusp (hypocone) showed the highest average variation, with a value of 0.0159, indicating that this region of the molars exhibits the greatest morphological difference between contralateral teeth. The mesiolingual cusp (protocone), which had an average variation of 0.0079, showed a moderate level of difference.

In addition to these comparisons, we aimed to explore the potential differences in the similarity of contralateral molars within each group, i.e., males and females (Figure 3). The Euclidean distances between the left and right molars of each individual were calculated separately for males and females to assess whether the degree of intra-individual similarity differed between the two sexes. Before performing the comparison, the normality of the distance distributions for both males and females was evaluated using the Shapiro-Wilk test. The data for males followed a normal distribution (*p* = 0.784), while for females, the distribution was non-normal (*p* = 0.046). Levene’s test showed no significant difference in the variances between the two groups (*p* = 0.727), allowing us to proceed with a two-sample *t*-test. The mean distance for females was 0.0317, while for males, it was 0.0383. The *t*-test (t = 1.009, *p* = 0.320) revealed no statistically significant difference between the two groups, indicating that the similarity between contralateral molars does not differ significantly between males and females.

## 4. Discussion

The primary aim of this study was to evaluate, using a new digital approach (GMM), the applicability and precision in detecting the features that compose the dental crowns of first upper molars. The goal was to highlight the consistency and similarity of contralateral teeth, including their crown morphology, and to identify any areas of distinction in a sample of Italian individuals. The analysis revealed that molars from opposite sides (left and right) within the same individual exhibit significantly greater morphological similarity compared to molars from different individuals. Specifically, the Euclidean distances between contralateral molars were smaller (mean = 0.035) than those observed between molars from different individuals (mean = 0.052). These findings confirm the hypothesis that contralateral molars share a high degree of morphological congruence, likely due to the shared genetic and developmental pathways, as previously suggested in the literature [20].

This intra-individual stability in dental morphology is particularly evident in the occlusal surfaces, which play a critical role in chewing efficiency. The arrangement of cusps, grooves, and fossae was highly consistent between left and right molars, ensuring proper occlusion and functional harmony. These results align with earlier studies that highlighted the importance of geometric consistency in occlusal morphology for maintaining an efficient masticatory function [21]. Even minor deviations in occlusal morphology can negatively impact chewing efficiency, underscoring the functional importance of this morphological stability.

Although slight differences were observed between contralateral molars—such as minor variations in the palatal cusps (hypocone and protocone)—these likely reflect natural biological variability, including subtle developmental asymmetries. Such variations are expected within the context of normal dental development and do not detract from the overall high degree of congruence observed.

Moreover, this morphological stability becomes especially important when considering the functional relationship between the upper and lower molars, particularly in occlusion (for example, [22]). The palatal cusps of the upper first molar, which show a slight variability within the sample analyzed by us, come into occlusion with the buccal cusps of the lower first molar. As noted in various studies [10,23,24], the lower molar tends to have a thicker enamel layer, particularly in the buccal region. This thicker enamel likely serves as a biomechanical adaptation to withstand the forces generated during mastication, while compensating for the morphological variability in the upper molar’s palatal cusps. The enamel distribution on the lower molar may act as a buffer, accommodating slight variations in occlusal morphology [25] and maintaining efficient chewing dynamics [26,27]. This interplay between the upper and lower molars supports the idea that enamel thickness is not solely a reaction to mechanical stress, but also an adaptive feature that accommodates the morphological variability seen in antagonistic teeth. Specifically, the thicker enamel in the buccal region of the lower molar compensates for both the increased mechanical loads it endures and the potential variability in occlusal contact caused by differences in the upper molar’s palatal morphology. This suggests a complex relationship between form and function, where genetic and functional factors work together to ensure the overall stability of dental structures and effective mastication despite individual variations.

In contrast, molars from different individuals showed significantly greater morphological variation, suggesting that molar morphology is highly individualized, shaped by genetic diversity and developmental asymmetries [9,28,29]. The variation observed in occlusal topography, including the depth and complexity of grooves and fossae, underscores the influence of individual-specific factors in dental development. This further highlights how dental morphology, while generally stable within individuals, can vary considerably between individuals, reflecting both genetic variability and environmental pressures.

### 4.1. Implications for Dentistry and Dental Prosthetics

The high degree of morphological similarity between contralateral molars has significant implications for restorative dentistry and dental prosthetics. In clinical settings, accurately replicating a patient’s natural tooth morphology is crucial for maintaining functional occlusion and ensuring comfort. Our findings suggest that contralateral molars can serve as reliable anatomical templates for dental restorations. When a molar is damaged or missing, dental technicians can use the contralateral molar as a model for fabricating prostheses that closely match the patient’s natural tooth. This approach ensures a better occlusal fit, functional integration, and overall patient satisfaction [30].

However, when using the contralateral molar as a template for dental reconstruction, special attention must be paid to the degree of dental wear it may exhibit. While the morphology of contralateral molars tends to be highly similar, intra-individual variations in wear can occur due to the differences in occlusal dynamics between the right and left sides. These differences can generate distinct wear patterns that need to be accounted for. To address this, careful observation of the opposing (antagonist) tooth is crucial. The antagonist can serve as a guide to modify and adapt the occlusal morphology of the reconstructed tooth, ensuring it makes optimal contact with the opposing molar, and thus, restoring function as closely as possible to the original.

Furthermore, the importance of a personalized approach in prosthodontics is highlighted by the morphological differences observed between individuals. In fact, the standardization of methodologies is proposed by authors such as Peter Thomas, Richard Landin, Rudolf Slavicek, Gerhard Polz. These methodologies have enabled the standardized reproduction of dental structures, facilitating the fabrication of dental prostheses, while maintaining a personalized approach to meet individual patient needs.

However, these methods do not adequately account for the variability of natural dental morphology, which is strongly influenced by genetic and individual factors. For example, Slavicek primarily focuses on the interaction between occlusion and the temporomandibular joint (TMJ), emphasizing TMJ functionality over tooth morphology [31]. Polz, on the other hand, bases his approach on the vertical dimension of occlusion, paying particular attention to the correct occlusal height to maintain masticatory balance [32]. Landin emphasizes muscular and joint stability, seeking functional harmony between masticatory muscles and prostheses [33]. Thomas focuses on analyzing mandibular movements to prevent occlusal dysfunctions, designing prostheses based on mandibular dynamics [34].

While these tools offer a high degree of customization, they cannot fully capture the natural dental morphology, which may present individual variations. Such variations include morphological discrepancies between contralateral teeth, the presence of non-metric traits, and dental wear, all of which must be considered to ensure proper occlusion. As highlighted by this study, dental reconstruction requires subjective consideration, as tooth shapes are genetically predetermined [35].

However, the increasing integration of digital technologies now allows us to overcome these limitations by more precisely tailoring prosthetics to individual morphological variability. Our results, derived from studying the shape of the upper first molar, suggest that significant morphological differences among molars from different individuals make a standardized approach inadequate. Furthermore, attempting to establish a numerical relationship between all teeth using standardized methods or neutral approaches, which rely on “one-size-fits-all” tooth libraries, requires subsequent adjustments—whether through virtual corrections or traditional manual methods—to achieve proper customization. By utilizing our approach, it is possible to capture patient-specific anatomical details, allowing for the creation of customized prostheses, crowns, and implants that align with the individual’s dental anatomy. This personalized approach is particularly critical for complex occlusal surfaces, where even small deviations from natural morphology could result in functional issues, such as poor occlusal fit, discomfort, or inefficient mastication.

### 4.2. Implications in Evolutionary Anthropology

The finding that contralateral molars are morphologically similar but exhibit some variability, particularly in the lingual surface, including the protocone and the hypocone cusps, carries important implications for morphological interpretations in evolutionary anthropology. Teeth are often the most common fossil remains of both human and non-human ancestors, and they play a critical role in species identification and evolutionary analysis [36,37]. However, this study suggests that relying on the morphology of a single tooth—often the case when only one dental specimen is available—may lead to incomplete or even misleading interpretations of species variability [38].

In evolutionary contexts, identifying species based solely on the dental morphology of one individual can underestimate the natural variation that exists within a population. As shown in this study, even within the same individual, there is a degree of morphological variability between contralateral teeth, albeit less pronounced than inter-individual differences. This intra-individual variation can complicate taxonomic classification, especially when fossil remains are fragmentary or limited to a few teeth [39]. Moreover, the recognition of intra-individual variability emphasizes that a single tooth may not fully represent the dental morphology of an entire species. Evolutionary anthropologists must account for this variability to avoid oversimplifying the morphological diversity within a given population or species.

By integrating a more nuanced understanding of dental variability, particularly regarding specific cusps like the protocone and hypocone, researchers can refine their interpretations of fossil remains, leading to more accurate reconstructions of evolutionary lineages and better identification of hominin species. This insight also underscores the importance of using multiple specimens when available, rather than relying on isolated teeth for species identification. Achieving this will enhance the accuracy of evolutionary models and ensure that the full range of morphological variations within a species are properly documented. The variability within dental structures is not merely an artifact of wear or damage, but may reflect deeper evolutionary adaptations, influenced by both genetic and environmental factors (for example, [10,40]).

Although this study is limited by its relatively small sample size, the method demonstrates significant potential to revolutionize the precision of metric data quantification in medical applications. This approach could greatly enhance the accuracy of morphological analyses, paving the way for advancements in personalized and precision medicine.

## 5. Conclusions

This study demonstrates how the geometric morphometric analysis is an effective tool for identifying the remarkable morphological similarity between contralateral molars within the same individual. This methodological approach enabled the precise quantification of dental morphology, highlighting the potential of using contralateral molars as reliable anatomical templates for prosthetic individualized reconstructions. At the same time, the significant variability observed between molars from different individuals emphasizes the importance of incorporating personalized data to ensure accurate and functionally integrated dental restorations.

## Data Availability

The original contributions presented in this study are included in the article. Further inquiries can be directed to the corresponding author.
